# Survival Outcomes Among Patients With High-Grade Glioma Treated With 5-Aminolevulinic Acid–Guided Surgery: A Systematic Review and Meta-Analysis

**DOI:** 10.3389/fonc.2019.00620

**Published:** 2019-07-17

**Authors:** Sirin Gandhi, Ali Tayebi Meybodi, Evgenii Belykh, Claudio Cavallo, Xiaochun Zhao, Masood Pasha Syed, Leandro Borba Moreira, Michael T. Lawton, Peter Nakaji, Mark C. Preul

**Affiliations:** ^1^Department of Neurosurgery, St. Joseph's Hospital and Medical Center, Barrow Neurological Institute, Phoenix, AZ, United States; ^2^Department of Neurosurgery, Irkutsk State Medical University, Irkutsk, Russia; ^3^Department of Medicine, Saint Vincent Hospital, Worcester, MA, United States

**Keywords:** 5-aminolevulinic acid, fluorescence-guided surgery, glioblastoma multiforme, gross total resection, high-grade glioma, meta-analysis, survival outcome

## Abstract

**Background:** High-grade glioma (HGG) is associated with a dismal prognosis despite significant advances in adjuvant therapies, including chemotherapy, immunotherapy, and radiotherapy. Extent of resection continues to be the most important independent prognosticator of survival. This underlines the significance of increasing gross total resection (GTR) rates by using adjunctive intraoperative modalities to maximize resection with minimal neurological morbidity. 5-aminolevulinic acid (5-ALA) is the only US Food and Drug Administration–approved intraoperative optical agent used for fluorescence-guided surgical resection of gliomas. Despite several studies on the impact of intra-operative 5-ALA use on the extent of HGG resection, a clear picture of how such usage affects patient survival is still unavailable.

**Methods:** A systematic review was conducted of all relevant studies assessing the GTR rate and survival outcomes [overall survival (OS) and progression-free survival (PFS)] in HGG. A meta-analysis of eligible studies was performed to assess the influence of 5-ALA-guided resection on improving GTR, OS, and PFS. GTR was defined as >95% resection.

**Results:** Of 23 eligible studies, 19 reporting GTR rates were included in the meta-analysis. The pooled cohort had 998 patients with HGG, including 796 with newly diagnosed cases. The pooled GTR rate among patients with 5-ALA–guided resection was 76.8% (95% confidence interval, 69.1–82.9%). A comparative subgroup analysis of 5-ALA–guided vs. conventional surgery (controlling for within-study covariates) showed a 26% higher GTR rate in the 5-ALA subgroup (odds ratio, 3.8; *P* < 0.001). There were 11 studies eligible for survival outcome analysis, 4 of which reported PFS. The pooled mean difference in OS and PFS was 3 and 1 months, respectively, favoring 5-ALA vs. control (*P* < 0.001).

**Conclusions:** This meta-analysis shows a significant increase in GTR rate with 5-ALA–guided surgical resection, with a higher weighted GTR rate (~76%) than the pivotal phase III study (~65%). Pooled analysis showed a small yet significant increase in survival measures associated with the use of 5-ALA. Despite the statistically significant results, the low level of evidence and heterogeneity across these studies make it difficult to conclusively report an independent association between 5-ALA use and survival outcomes in HGG. Additional randomized control studies are required to delineate the role of 5-ALA in survival outcomes in HGG.

## Introduction

High-grade gliomas (HGGs), including World Health Organization (WHO) grade III and grade IV gliomas, are the most common subtype of primary adult malignant cerebral neoplasms with a poor prognosis. Current management strategies include maximal safe surgical resection along with a combination of chemotherapy and fractionated radiotherapy. Complete resection of enhancing tumor (CRET) or gross total resection (GTR) have been demonstrated to be independent prognosticators of increased progression-free survival (PFS) and overall survival (OS) (1). However, true delineation of the tissue boundaries of HGGs is extremely difficult. Gadolinium enhancement on magnetic resonance imaging (MRI) is conventionally used to determine the tumor boundaries and postoperative resection analysis. In recent years, fluorescence image-guided resection using agents like fluorescein sodium (2), indocyanine green (3, 4), and 5-aminolevulinic acid (5-ALA) (5, 6) has gained significant traction in the treatment of HGGs. These optical agents can augment visual differentiation of the tumor border zone intraoperatively by selective fluorescence of the abnormal diseased tissue (7, 8).

5-ALA is currently approved in many countries, with US Food and Drug Administration (FDA) approval obtained a decade after European Medical Agency (EMA) approval (9). Selective accumulation of protoporphyrin IX (PpIX), which is an endogenous fluorophore and a downstream metabolite of 5-ALA in the heme synthesis pathway, occurs in the malignant glioma cells in HGGs (10). PpIX-fluorescence is best visualized using filtered xenon light with blue-violet light 375–440 nm wavelength and an emission filter for red fluorescence of 635–704 nm (8, 11). The standard dose of 5-ALA oral administration is 20 mg/kg dissolved in 50 mL of water given 3 h prior to surgery with a peak visible fluorescence at 6–8 h after consumption (12). However, photobleaching effects have been observed in this tumor-specific fluorescence, with 36% fluorescence decay in 25 min at an excitation wavelength of 405 nm and after 87 min under unfiltered wide-field illumination (8, 13).

Several studies have suggested that intraoperative PpIX accumulates even in the absence of contrast enhancement on MRI and supersedes the margins of contrast-enhancing tumor in the gadolinium-enhancing tumor subtypes (14, 15). Thus, it is imperative to determine the added surgical benefit of resection of this residual non-enhancing fluorescent tissue while minimizing any postoperative neurological morbidity. This particular question was examined in 50 patients with GBM who underwent operations with 5-ALA-based fluorescence guidance with matched non-surgical prognostic markers, such as age, location, O^6^-methylguanine-DNA-methyltransferase status, and Karnofsky Performance Scale score (14). The mean OS was significantly worse in the CRET subgroup (17 months; 95% confidence interval [CI]: 22.4–31.6 months) if patients had persistent residual fluorescence in comparison with maximal safe fluorescent tissue resection (27 months; 95% CI: 12.5–22.5 months) (14). This study demonstrated discrepancy between intraoperative tissue fluorescence and gadolinium contrast enhancement. Use of adjunctive tools like navigation with tractography, somatosensory evoked potential or motor evoked potential mapping, confocal endomicroscopy, intraoperative MRI (iMRI), ultrasound, or awake craniotomy may be beneficial in conjunction with fluorescence-guided surgical resection of aggressive HGGs in eloquent locations. In the current age of personalized and precision medicine, 5-ALA seems to have been identified as a tool in surgical theranostics, with benefits seen during photodynamic therapy using 5-ALA in experimental studies and patients with glioma (16–18).

The current standard of care for HGGs, including GBM, includes the Stupp protocol, which calls for maximal safe surgical resection with concomitant temozolomide and radiation therapy (19). The largest systematic review to date with quantitative analysis of the influence of extent of resection (EOR) on survival outcomes in patients with GBM observed a substantial improvement in PFS and OS in patients undergoing GTR (1). Since 5-ALA has been shown to significantly improve EOR and GTR/CRET in patients with HGG, this would indirectly imply an enhanced survival benefit for this subset of patients. However, there is a paucity of quantitative data analysis in reported systematic reviews owing to the heterogeneity of the patient population and low levels of scientific evidence based on study designs (10, 20–22).

Despite the previous systematic reviews assessing the value of 5-ALA for the treatment of HGG (10, 20, 21), the benefit of 5-ALA to patients (e.g., improvements in neurological outcomes, quality of life, and overall survival) remain inconclusive. The benefit of 5-ALA has so far been demonstrated only indirectly by showing increased GTR rates when using fluorescence guidance. We attempted to bridge the gap and investigate the evidence for 5-ALA benefits on the basis of patient outcome–based metrics. The objective of this study was to conduct a systematic review of the literature and perform a quantitative pooled evaluation of survival outcomes and GTR rates of 5-ALA–guided surgical resection in HGGs. Since our pooled cohort included patients with recurrent HGG, a subgroup outcomes analysis among patients with primary HGG was also performed to assess the survival outcomes.

## Methods

This systematic review was conducted in accordance with the Preferred Reporting Items for Systematic Reviews and Meta-Analyses (PRISMA) guidelines. This study was exempt from institutional review board approval as it was a meta-analytical review of literature.

### Search Strategy

PubMed, MEDLINE, and EMBASE electronic databases were searched to identify studies from the database inception through December 31, 2018, regarding the use of intraoperative 5-ALA for fluorescence-guided resection of HGGs. No studies were excluded on the basis of publication type or date of publication. However, preclinical and *in vitro* studies involving animals were excluded. MeSH and EMTREE terms relevant to 5-ALA and HGGs were used to search the PubMed, MEDLINE, and EMBASE databases. These terms included “aminolevulinic acid,” “levulinic acid,” “5ALA” or “5-ALA,” “glioma,” “high grade glioma,” “malignant glioma,” and “glioblastoma.” The bibliography of all articles meeting the inclusion criteria was examined to identify any missing additional studies. All articles from this search were added to an Endnote library X 8.2 (Clarivate Analytics, Philadelphia, PA). Non-English literature was excluded from this study.

### Systematic Review Protocol and Data Extraction

Titles and abstracts were screened by two independent reviewers (SG, ATM) with input from the senior author (MCP) in case of ambiguity. Inclusion criteria for this meta-analysis were as follows: (1) all patients with histopathologically confirmed (WHO grade III and grade IV) HGG including recurrent gliomas and (2) all studies reporting on the GTR or CRET rate and survival outcomes (OS and PFS) pertinent to the use of 5-ALA in the surgical resection of HGGs in adult patients. Laboratory studies, technical reports, review articles including systematic reviews, editorials, commentaries, conference proceedings, and abstracts were excluded from the analyses. Studies were also excluded if there was a lack of clear reporting of HGG type (i.e., whether HGG was primary or recurrent), if they had a small sample size (*n* < 10), if they included overlapping patient populations, and if we were unable to extract analyzable data points.

Full-text review of the studies shortlisted using the above methodology was conducted by two reviewers in an independent fashion, and the relevant data were subsequently extracted from each study for further statistical analyses. The variables of interest included first-author name, year of publication, study design, sample size, demographic distribution, follow-up duration, survival data, EOR, and subtypes and location of glioma. The study design was used as a surrogate marker for assessing any biases. Studies were analyzed quantitatively for three major parameters: GTR (defined as >95% tumor volume resection), PFS, and OS rates. In studies that reported a 95% CI and no standard deviation (SD) in their results, the SD value was obtained from a formula using the standard errors and 95% CI values as follows:

$$\text{SD}=\;\sqrt{N}\times \frac{\left(\text{UL-LL}\right)}{\text{3}.\text{92}}$$

where *N* is the number of subjects, UL is the upper limit and LL is the lower limit of the 95% CI.

Additionally, in studies with a lower sample size (*n* < 50), the denominator of 3.92 was replaced with a manual *t* distribution calculation using the degree of freedom (23). This calculation allowed for inclusion of a few studies in our final analyses that have been excluded in the previously reported studies.

### Statistical Analysis of Data

After obtaining the proportional values of GTR >95% and the mean (SD) values for OS and PFS as described above, the statistical analysis was conducted using an open-source meta-analytical software, Open MetaAnalyst for Mac OS Sierra 10.12 (Brown University, Providence, RI). Heterogeneity across the various studies included for the meta-analyses was determined using an *I*^2^ statistical test for inconsistency with an arbitrary cutoff value of *I*^2^ ≥ 50% with a *P* < 0.05 representing significant heterogeneity. A fixed-effect model was used for conducting the meta-analysis in the absence of substantial heterogeneity, and a random-effect model was used for significant heterogeneity across the various combined studies. The random-effect model utilized the DerSimonian-Laird method to evaluate within-study variance (24).

## Results

**Figure 1:**
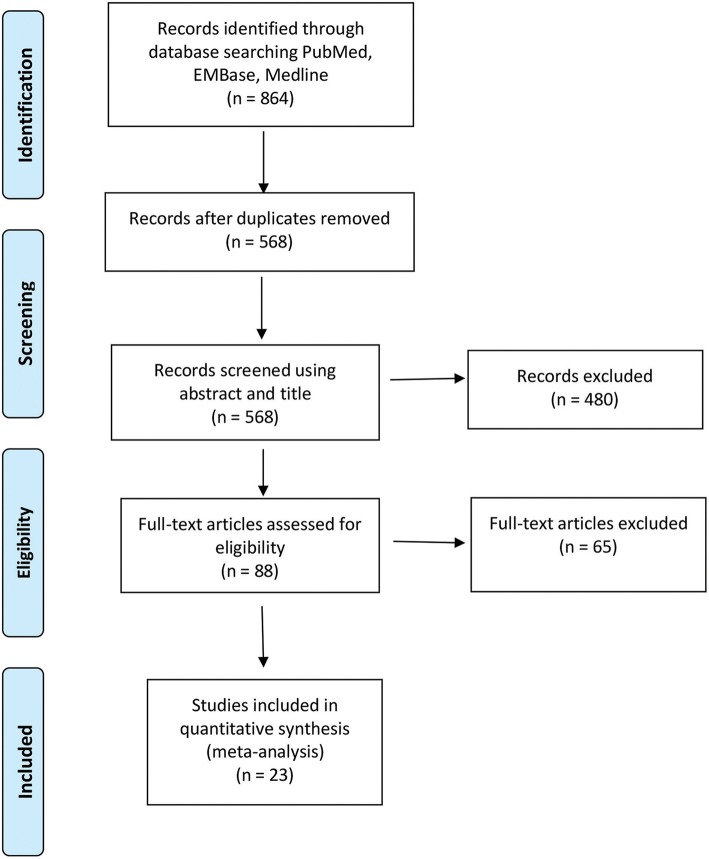
Preferred Reporting Items for Systematic Reviews and Meta-Analyses (PRISMA) flow diagram of the studies included in the meta-analysis of resection and survival outcomes among patients with 5-aminolevulinic acid–guided resection of high-grade glioma. Used with permission from Barrow Neurological Institute, Phoenix, Arizona.

The initial screening using the search strategy described in the Methods section yielded 864 articles, commentaries, and conference proceedings. After removal of duplicates and screening for non-English and non-relevant articles, 88 full-text articles remained. Each full-text article was checked by two independent reviewers to confirm that it met the inclusion criteria (Figure 1). Sixty-five articles were excluded because they did not meet the aforementioned eligibility criteria for this study. Twenty-three studies were included in the final meta-analysis of the pooled GTR and survival outcomes analysis.

### 5-ALA and Gross Total Resection

**Figure 2:**
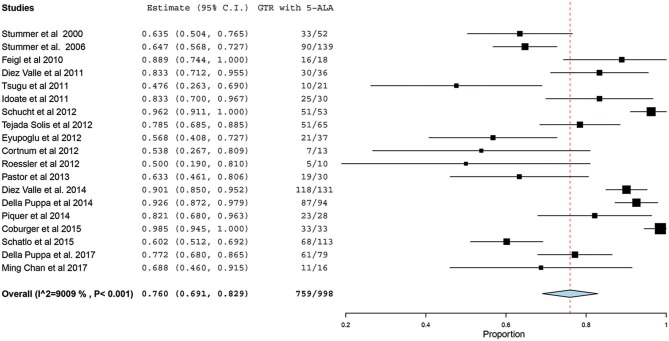
Forest plot graph representing the meta-analysis of gross total resection (GTR) rate (95% confidence interval [CI]) among patients with high-grade glioma, including primary and recurrent cases, treated using 5-aminolevulinic acid (5-ALA)–guided surgical resection. Used with permission from Barrow Neurological Institute, Phoenix, Arizona.

**Figure 3:**
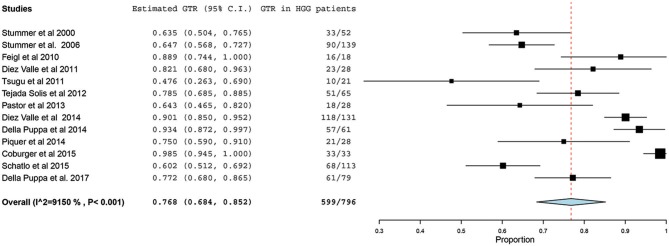
Forest plot graph representing the meta-analysis of gross total resection (GTR) rate (95% confidence interval [CI]) among patients with primary high-grade glioma (HGG) treated using 5-aminolevulinic acid–guided surgical resection. Used with permission from Barrow Neurological Institute, Phoenix, Arizona.

Of the 23 studies included in the analyses, 19 reported a GTR or CRET rate (5, 25–42). The number of participants who underwent 5-ALA–guided resection in these studies ranged from 10 to 139, with the GTR ranging from 47.6 to 98.5%. The *I*^2^ statistics showed a significant heterogeneity level (90.09%; *P* < 0.001). Therefore, a random-effect model was used for the pooled estimate of these data. In pooled analysis of all HGGs, including primary and recurrent subtypes, GTR was achieved in 759 of 998 patients, resulting in an overall GTR rate of 76% (95% CI, 69–83%; *P* < 0.001) (Figure 2). In the subgroup analysis of primary GBM alone, GTR was achieved in 599 of 796 patients, resulting in a similar GTR rate of 76.8% (95% CI, 68–85%, *P* < 0.001) (Figure 3).

**Figure 4:**
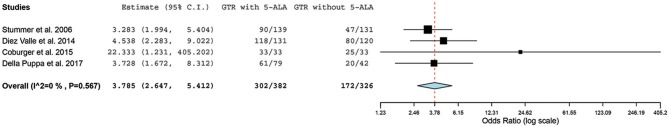
Forest plot graph representing the meta-analysis of the odd ratio (95% confidence interval [CI]) of gross total resection (GTR) among patients with primary high-grade gliomas treated using 5-aminolevulinic acid (5-ALA)–guided surgical resection vs. conventional microsurgical resection. Used with permission from Barrow Neurological Institute, Phoenix, Arizona.

Four case-controlled studies were found to be eligible for a meta-analysis of GTR with 5-ALA (*n* = 382) vs. conventional resection (*n* = 326) (31, 34, 37, 43). All other covariates (e.g., use of intraoperative MRI, postoperative treatment with specific chemotherapeutic agents) remained the same within each study but were variable across the studies. However, there was no significant statistical heterogeneity across these studies (*I*^2^ = 0%; *P* = 0.57); hence, we used a fixed-effect model for analysis. GTR was achieved in 302 of 382 (79.1%) patients in the 5-ALA group vs. 172 of 326 (52.8%) patients in the control group. In the pooled estimate of all HGGs (primary and recurrent), 5-ALA was associated with a 26% increase in GTR rate compared with the control group in which surgeries were performed without 5-ALA, resulting in OR = 3.79 (95% CI, 2.65–5.41; *P* < 0.001) for achieving GTR (Figure 4).

### Overall Survival Data for 5-ALA–Guided Glioma Resection

**Figure 5:**
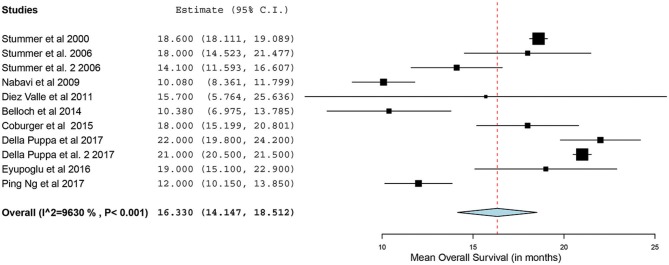
Forest plot graph representing the meta-analysis of mean overall survival (95% confidence interval [CI]) among patients with primary high-grade gliomas treated using 5-aminolevulinic acid–guided surgical resection. Used with permission from Barrow Neurological Institute, Phoenix, Arizona.

**Figure 6:**
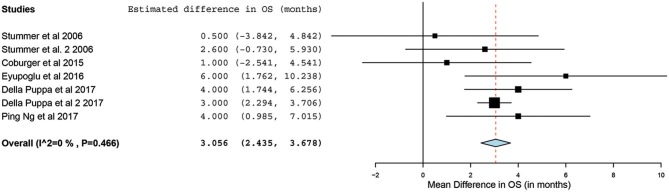
Forest plot graph representing the meta-analysis of mean difference in overall survival (95% confidence interval [CI]) among patients with primary high-grade glioma treated using 5-aminolevulinic acid–guided surgical resection vs. conventional microsurgical resection. Used with permission from Barrow Neurological Institute, Phoenix, Arizona.

**Figure 7:**
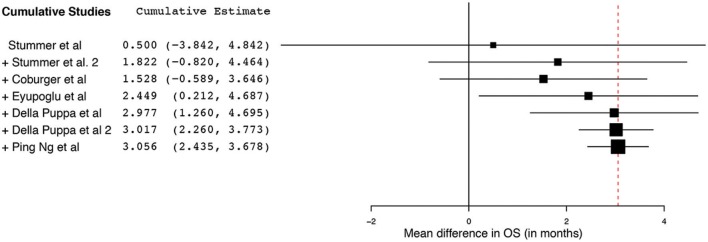
Cumulative regression analysis demonstrating the temporal trend of mean difference (95% confidence interval [CI]) in overall survival among patients with primary high-grade glioma treated using 5-aminolevulinic acid–guided surgical resection vs. conventional microsurgical resection. Used with permission from Barrow Neurological Institute, Phoenix, Arizona.

Of the 23 studies, 9 reported a mean OS period in months ranging from 10 to 22 months (5, 25, 26, 31, 34, 44–47). One of the studies, by Stummer et al. (31), stratified the survival analysis by age (55 years of age and younger vs. older than 55 years). Hence, these patients were included in our analysis as two independent patient groups conducted by the same methodology used by this phase III randomized control trial (RCT) (31). Similarly, another study had subdivided its patients into two treatment groups (treated using 5-ALA) and one control group (treated without the use of 5-ALA).Thus, we treated the two treatment arms as independent patient groups in our analysis for OS (25). Therefore, 11 distinct groups of patients who underwent 5-ALA–guided resection of primary HGG were extracted for analysis. There was significant heterogeneity across these groups (*I*^2^ = 96.3%; *P* < 0.001); therefore, a random-effects model for continuous variable was used. The meta-analysis in the group of patients with primary HGG who underwent 5-ALA–guided resection showed a mean OS of 16.3 months (95% CI, 14.1–18.5 months; *P* < 0.001) (Figure 5). Five studies (seven distinct 5-ALA groups) were available for comparison of OS (measured as a mean survival difference) between 5-ALA vs. control groups (25, 31, 34, 46, 47). No significant heterogeneity was noted across these groups (*I*^2^ = 0%; *P* = 0.47); hence, we used a fixed-effects model. The meta-analysis yielded a pooled OS difference of 3.05 months (95% CI, 2.43–3.68 months; *P* < 0.001) favoring 5-ALA-guided resection compared with control groups without 5-ALA (Figures 6, 7).

### Progression Free Survival Data for 5-ALA Guided Glioma Resection

**Figure 8:**
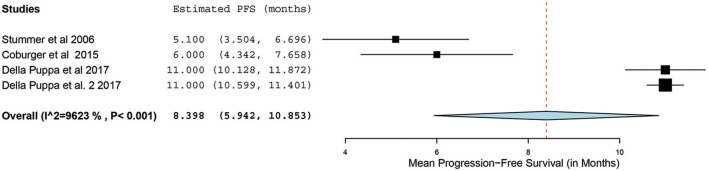
Forest plot graph representing the meta-analysis of mean progression-free survival (95% confidence interval [CI]) among patients with primary high-grade glioma treated using 5-aminolevulinic acid–guided surgical resection. Used with permission from Barrow Neurological Institute, Phoenix, Arizona.

**Figure 9:**
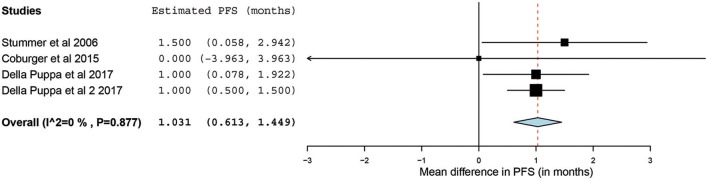
Forest plot graph representing the meta-analysis of mean difference in progression-free survival (95% confidence interval [CI]) among patients with primary high-grade glioma treated using 5-aminolevulinic acid–guided surgical resection vs. conventional microsurgical resection. Used with permission from Barrow Neurological Institute, Phoenix, Arizona.

**Figure 10:**
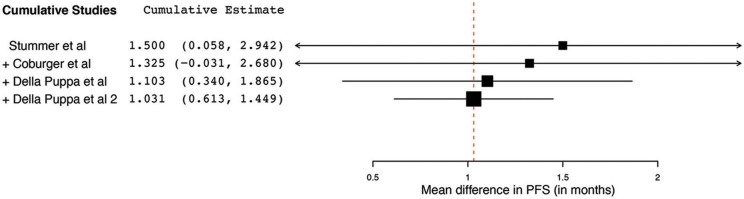
Cumulative regression analysis demonstrating the temporal trend of mean difference in progression-free survival (95% confidence interval [CI]) among patients with primary high-grade glioma treated using 5-aminolevulinic acid–guided surgical resection vs. conventional microsurgical resection. Used with permission from Barrow Neurological Institute, Phoenix, Arizona.

Of the 23 papers, three studies reported PFS ranging from 5.1 to 11 months. From these 3 studies, 4 distinct groups of patients who underwent 5-ALA–guided resection of primary HGG were extracted for analysis. Significant heterogeneity was noted across these groups (*I*^2^ = 96.3%; *P* < 0.001), with use of a random-effects model for continuous variables. The meta-analysis of patients with primary HGG who underwent 5-ALA–guided resection showed a mean PFS of 8.4 months (95% CI, 5.9–10.9 months; *P* < 0.001) (Figure 8). The mean PFS difference was 1.03 months (95% CI, 0.61–1.45 months; *P* < 0.001) favoring 5-ALA when compared with matched control groups (Figures 9, 10).

## Discussion

### Interpretation of the Meta-Analytic Data

This meta-analysis was conducted to evaluate the potential survival advantage gained, if any, with the use of 5-ALA–guided resection of HGGs. The pooled analysis of 998 patients with HGG who underwent 5-ALA–guided surgical resection showed a higher rate of GTR in the cohort of all patients with HGG (~76%) as well as in the subgroup of patients with primary HGG (~77%), as compared with the phase III RCT conducted by Stummer et al. (31) that reported a GTR rate of 65%. The pooled analysis of the eligible case-controlled studies demonstrated that the estimated GTR rate was significantly higher with the intraoperative use of 5-ALA compared with conventional white light technique surgery (79 vs. 53%; OR = 3.78; *P* < 0.001). As GTR is the most significant independent prognostic marker of survival in patients with HGG (1), these results encourage the utilization of fluorescence-guided resection of these lesions. There was a high level of heterogeneity across the studies included in this pooled GTR analysis, attributable to the observational nature of some of these studies and a multitude of other factors that are difficult to control for. For example, invasion of eloquent cortical tissue by an aggressive lesion, determined by preoperative imaging or intraoperatively, is a critical reason for planned subtotal resection, irrespective of the use of 5-ALA. However, inclusion of such preplanned subtotal resection can contribute to an underestimation of the rate of GTR truly attributable to the use of fluorescence guidance. Unfortunately, owing to the lack of specific data in these studies regarding GTR rate in eloquent cortical locations, an analysis could not be performed on this specific population.

The collated survival analysis demonstrated an overall survival advantage (~3-months higher mean OS; *P* < 0.001) with the use of 5-ALA. This has been represented using a cumulative meta-analysis that shows a positive trend in the increase in mean survival difference across the studies published over time (Figure 7). However, OS is not the most convenient study endpoint owing to the vast heterogeneity and lack of control on the various postoperative adjuvant therapies that can influence the overall survival. Additionally, in recent years, there seems to be an increase in survival for all patients with HGG that is perhaps attributable to the standardization of the Stupp protocol (19), constant evolution of chemotherapeutic agents and radiotherapy modalities, and introduction of immunotherapy and targeted genetic therapies (48, 49). PFS is a better primary study endpoint for survival analysis than OS because of shorter follow-up period, controlling for the effects of non-study-related therapies in influencing survival and avoiding confusion from the multiple reasons for death. Unfortunately, the amount of data available on PFS in controlled studies is limited, as shown by the cumulative meta-analysis (Figure 10). Of the three studies included in PFS analysis, a statistically significant delay of 1 month in time to progression was noted in our pooled cohort (25, 26, 31, 34).

### Evolution of Evidence on Benefits of 5-ALA–Guided HGG Surgery

The first clinical study on 5-ALA was reported in 1998, which led to the development of the pivotal randomized, multicenter phase III clinical trial in 5-ALA by Stummer et al. (31) in 2006. The study reported a 29% difference in CRET (65% in 5-ALA vs. 36% using white light surgery; *P* < 0.001). This study demonstrated superior clinical outcomes, significantly higher rates of CRET, and significant improvement in PFS at 6 months using fluorescence-guided surgery vs. the conventional wide-field microsurgical resection (31). In 2007, on the basis of the results of the aforementioned trial, EMA approved the use of 5-ALA. Thereafter, the drug was approved by various countries. In 2011, an FDA request for approval of 5-ALA was filed. The conceptualization of this drug as a therapeutic agent rather than an intraoperative diagnostic tool led to significant delay in the approval of 5-ALA in the United States (9). The drug received FDA approval in June 2017 and became the first optical agent approved by the FDA for use as an intraoperative imaging resource for the resection of HGGs. It is critical to treat 5-ALA and other optical agents as diagnostic markers or tools and not as a means of therapeutic intervention. 5-ALA fluorescence guidance is a modality that is associated with a direct benefit of improving GTR rates and an indirect effect on survival outcomes by increasing maximal safe surgical resection. This is the reason our study chose to assess GTR rates and survival outcomes (OS, PFS) in our final analysis. Concurrently, since the early 2000s, various preclinical and a few clinical studies have investigated the potential therapeutic role of 5-ALA for photodynamic therapy for malignant gliomas with what appear to be advantageous preliminary results (16, 18, 50–59).

### Previous Systematic Reviews and Meta-Analyses on 5-ALA–Guided Surgical Resection

Several systematic literature reviews have evaluated the role of fluorescence-guided surgery grouping several optical agents (e.g., fluorescein, 5-ALA, indocyanine green) in terms of GTR, diagnostic metrics (sensitivity and specificity), and survival outcomes (18, 21, 22, 60, 61). Because fluorescein, 5-ALA, and indocyanine green have substantially different fluorescent properties and mechanistic behaviors, our study focused on the evaluation of these important study endpoints with the use of 5-ALA as a fluorophore. Eljamel et al. (20) conducted a meta-analysis of 5-ALA–guided resection of GBMs and reported a high sensitivity (82.6%) and specificity (88.9%). The 75% mean GTR rate was in congruence with our results that included a higher sample size. The mean PFS reported in that study was also similar to the PFS reported in our study (~8 months). In contrast with that study, we calculated the measures of continuous outcomes (e.g., SD) as mentioned in the Methods and included several observational studies that were excluded in the previous analyses.

Mansouri et al. (10) conducted a systematic review of literature to assess the adjunctive intraoperative role of 5-ALA in HGGs. The study included 43 articles (1 RCT, 28 prospective studies, and 14 retrospective studies) and concluded that 5-ALA was associated with improved tumor visualization with a diagnostic accuracy >80%, enabling a greater EOR, that could be indirectly linked to increased survival. Our study had similar inclusion criteria to the aforementioned study. However, we added the quantitative data analyses to objectively evaluate the added benefit of using 5-ALA in HGG surgery.

### Potential Limitations of 5-ALA

Fluorophores for intraoperative *in vivo* use in the brain are limited (62). Like other fluorophores in use, 5-ALA has certain drawbacks for its use in a clinical setting. Exogenous administration of 5-ALA leads to selective accumulation of PpIX in the malignant cells of HGG tumors, as mentioned previously (8, 11, 12). From a metabolomic perspective, the molecular mechanism of action of 5-ALA leading to a differential fluorescence of malignant cells can be attributed to the turnover rate of PpIX rather than its accumulation. For instance, in a study of WHO Grade III glioma patients with an *IDH-1* mutation, a depletion of nicotinamide adenine dinucleotide phosphate was hypothesized to mediate the differential temporary accumulation of PpIX (a downstream product of 5-ALA in heme synthesis pathway) in the malignant cells (63). Numerous studies have reported on the potential molecular mechanism for 5-ALA fluorescence properties in malignant cells (63–66). An in-depth analysis of this is beyond the scope of this article.

Blood brain barrier disruption is essential for the accumulation of PpIX in the malignant cells, which is hypothesized to be the cause for lower or negligible fluorescence in LGG cases (8). In addition, the visual PpIX fluorescence signal can be vague or inhomogeneous in appearance with various areas of high and low signal intensity even in HGGs (67, 68). This patchy appearance may be attributed to inhomogeneous tumor growth, glioma cell diversity, overall tissue component heterogeneity (e.g., cysts and necrotic tissue), and differential tumor cellular metabolism. Another level of complexity in accurate detection of fluorescence signal strength originates with the optical detection equipment (e.g., operating microscopes) (7). Limitations of wide-field operating microscopy imaging can, in turn, lead to reduced accuracy of tumor detection at the marginal infiltrative border, especially in eloquent cortical locations. Adjunctive tools that can improve fluorescence detection and provide intraoperative cellular or near-cellular resolution of PpIX fluorescence include the confocal laser endomicroscope (69) and the scanning fiber endoscope (67), whereas implicational or indirect indication of such fluorescent labeling can be correlated with spectroscopy (70–75) and other intraoperative diagnostic adjuncts, such as brain mapping, neuronavigation, ultrasound navigation, and intraoperative MRI (10, 41, 62, 75). These technologies, alone or synergistically, may help to alleviate these limitations and increase the overall EOR.

Photobleaching, defined as the degradation of fluorescence signal of PpIX with prolonged light exposure, is an important limiting phenomenon of which the neurosurgeon should be aware. PpIX photobleaching is directly related to the intensity of the operating microscope light as well as the duration of exposure (7). Photobleaching of PpIX is usually not of major concern during resection of high-grade gliomas, because new tissue layers with unbleached PpIX are exposed with the advancement of resection (11). However, photobleaching may impact accurate quantification or detection of any pathology with low fluorescence signal strength (7). Stummer et al. (11) and Belykh et al. (7) have discussed the potential reasons for insufficient or absent fluorescence and how to circumvent the technical issues, including but not limited to the presence of blood products or overlying soft tissue, photobleaching, improper 5-ALA administration, and incorrect microscope settings. Additionally, the use of 5-ALA requires intermittent switching between the blue light and the white light modes of the neurosurgical operating microscope. Use of 5-ALA likely increases operative time and certainly increases pharmaceutical procedure costs.

### Use of Intraoperative MRI in Conjunction With 5-ALA

In addition to neuronavigation and intraoperative cortical (sensory/motor) monitoring, several studies investigated the use of iMRI with 5-ALA guidance. As gadolinium enhancement on MRI is still the most reliable radiological marker of the EOR, these data are important to consider. Tsugu et al. (42) emphasized the utility of iMRI in patients with negative fluorescence, increasing the GTR to 55.6% from zero. However, the study did not show significant resection benefit with the adjunctive use of iMRI in patients with strong PpIX fluorescence signal. A retrospective study by Roder et al. (76) showed higher CRET rates in patients with iMRI plus 5-ALA vs. 5-ALA alone (74% vs. 45%; *P* = 0.02). Another study with 99 patients with malignant glioma undergoing 5-ALA–guided surgery in conjunction with iMRI reported a mean resection rate of 95% and suggested that 5-ALA allowed marginal tumor identification of the tumor extension beyond its radiological borders on iMRI (77). A recent systematic review by Coburger et al. concluded that the simultaneous use of 5-ALA in conjunction with iMRI can provide superior resection margins beyond radiological contrast enhancement (78). Obvious drawbacks of iMRI would be the added surgical time and significant increase in facilities and procedural costs. Although the assessment of iMRI in conjunction with 5-ALA was beyond the scope of this study, it may play a role in improving EOR and GTR in brain tumors with minimal or negative 5-ALA fluorescence.

### Potential Therapeutic Role of 5-ALA

Recent years have witnessed a surge in the institution of novel adjuvant therapies in the management of HGGs, including photodynamic therapy (18, 79). Photodynamic therapy causes direct cytotoxicity by activating cellular death via apoptosis, necrosis, autophagy, and paraptosis through various intrinsic intracellular mechanisms. Several *in vitro* and *in vivo* studies have evaluated the role of 5-ALA as a therapeutic agent for intraoperative photodynamic therapy of the residual tumor (50, 52, 57, 79). Early *in vivo* studies have shown promising results. Because 5-ALA is not cytotoxic on systemic administration and has a better adverse effect profile than the other photosensitizer therapies, it has the potential to function beyond its role as a diagnostic tool by becoming a part of the future armamentarium of adjuvant therapies for aggressive gliomas.

### Other Agents for Fluorescence Image-Guided Surgery

Apart from 5-ALA, fluorescein sodium has been used for intraoperative fluorescence-guided resection of HGGs. Fluorescein is an intravenous dye with selective accumulation through a damaged blood-brain barrier in malignant gliomas. A multicenter, controlled, Phase II trial showed complete resection of tumor in 82.6% patients with a 6-month PFS of 56.6%. However, the fluorescence characteristics of fluorescein are different than 5-ALA (2). Both exhibit variable fluorescence signal strength toward the periphery of the tumor, but, while 5-ALA stains cells, fluorescein is a non-specific dye leaked into the extravascular space by means of abnormal tumor vasculature or cortical trauma, with minor uptake by some cells (80). Thus, identification of the tumor marginal zone may be more difficult with fluorescein using wide-field fluorescence detection of the operating microscope, although it presents unique background staining of all cells visualized using confocal laser endomicroscopy (2, 67, 81). An advantage of fluorescein is that dosing can be repeated to a limited degree in the operating room. A pilot study recently investigated the synergistic use of 5-ALA and fluorescein in primary GBM. The complementary properties of these agents were used to capitalize on the advantages of each fluorophore to identify the tumor border zone in these patients (82). There are fewer studies on the use of fluorescein sodium compared to those using 5-ALA. Additional controlled comparative studies with a larger patient cohort are needed to establish the role of other fluorophores in image-guided resection of malignant gliomas.

### Study Limitations and Potential Biases

There have been a few publications that have systematically documented the various aspects of the role of 5-ALA in HGGs, as discussed above (10, 20–22). Our study focused on the quantitative data synthesis of EOR, OS, and PFS in HGGs. However, there is a high degree of heterogeneity in the data based on factors such as tumor location, radiological assessment of residual or recurrent tumor, different protocols for neoadjuvant or adjuvant therapy, differential resources or utilization of adjunctive intraoperative tools discussed above, and so on (37, 41, 46, 76). Additionally, the studies were performed at various institutions internationally, imparting a greater degree of diversity in management strategy. The level of evidence supporting the benefit of fluorescence-guided surgery using 5-ALA in HGG is very low to low quality in nature, with the exception of one RCT (31). Therefore, these data must be interpreted with caution. Since 5-ALA is already approved in most of the countries, conducting an additional controlled study might present an ethical problem. Further blinded multicenter RCTs will aid in generating class I level of evidence for the survival outcomes associated with 5-ALA and comparing 5-ALA to the other available intraoperative adjuncts (e.g., iMRI, ultrasound, confocal laser endomicroscopy, and scanning fiber endoscopy) to improve maximal safe EOR (8, 67, 83). Although we attempted careful selection of studies, we cannot control for the inherent limitations and potential risks of surgical trial and retrospective study biases in this pooled analysis of data.

## Conclusion

Our systematic review and meta-analysis show a modest survival benefit associated with the use of 5-ALA–guided surgery for HGGs. Unfortunately, at this time, the small number of studies and the low level of evidence of the available studies limit the ability to draw firm conclusions about the impact of 5-ALA–guided HGG resection on the OS and PFS of these patients. More high-quality studies with better controlling of confounding factors, such as adjuvant therapies, are needed to delineate the role of 5-ALA–guided surgery in improving survival of patients with these daunting tumors.

## Data Availability

The raw data supporting the conclusions of this manuscript will be made available by the authors, without undue reservation, to any qualified researcher.

## Author Contributions

SG: literature review and study selection, statistical analysis, figures, manuscript draft, revision, and final draft approval. AT and LB: manuscript draft, review of final draft. EB: manuscript draft, figures, review of final draft. CC: literature review and study selection, review of final draft. XZ: statistical analysis, review of final draft. MS: table for summary of studies, statistical analysis, review of final draft. ML, PN, and MP: study supervision, review and revision of final draft. MP: generation of the topic, approval of final draft.

### Conflict of Interest Statement

The authors declare that the research was conducted in the absence of any commercial or financial relationships that could be construed as a potential conflict of interest.

## Abbreviations

CRET: complete resection of enhancing tumor
EOR: extent of resection
FDA: US Food and Drug Administration
GBM: glioblastoma multiforme
GTR: gross total resection
HGG: high-grade glioma
iMRI: intraoperative magnetic resonance imaging
LGG: low-grade glioma
MRI: magnetic resonance imaging
OS: overall survival
PFS: progression-free survival
PpIX: protoporphyrin IX
RCT: randomized control trial
WHO: World Health Organization
5-ALA: 5 aminolevulinic acid.
